# Risk stratification in neuroblastoma patients through machine learning in the multicenter PRIMAGE cohort

**DOI:** 10.3389/fonc.2025.1528836

**Published:** 2025-02-21

**Authors:** Jose Lozano-Montoya, Ana Jimenez-Pastor, Almudena Fuster-Matanzo, Glen J. Weiss, Leonor Cerda-Alberich, Diana Veiga-Canuto, Blanca Martínez-de-Las-Heras, Adela Cañete-Nieto, Sabine Taschner-Mandl, Barbara Hero, Thorsten Simon, Ruth Ladenstein, Luis Marti-Bonmati, Angel Alberich-Bayarri

**Affiliations:** ^1^ Research & Frontiers in AI Department, Quantitative Imaging Biomarkers in Medicine, Quibim SL, Valencia, Spain; ^2^ Medical Studies Department, Quantitative Imaging Biomarkers in Medicine, Quibim Inc., New York, NY, United States; ^3^ Biomedical Imaging Research Group, La Fe Health Research Institute, Valencia,, Spain; ^4^ Pediatric Oncology and Hematology Section, La Fe University and Polytechnic Hospital, Valencia, Spain; ^5^ Sabine Taschner-Mandl Taschner-Mandl Group, St. Anna Children’s Cancer Research Institute, Vienna, Austria; ^6^ Department of Pediatric Oncology and Hematology, University Children’s Hospital of Cologne, Medical Faculty, University of Cologne, Cologne, Germany; ^7^ Clinical Trials Unit, St. Anna Children’s Cancer Research Institute, Vienna, Austria

**Keywords:** risk stratification, neuroblastoma, overall survival, pediatric, machine learning, PRIMAGE

## Abstract

**Introduction:**

Neuroblastoma, the most prevalent solid cancer in children, presents significant biological and clinical heterogeneity. This inherent heterogeneity underscores the need for more precise prognostic markers at the time of diagnosis to enhance patient stratification, allowing for more personalized treatment strategies. In response, this investigation developed a machine learning model using clinical, molecular, and magnetic resonance (MR) radiomics features at diagnosis to predict patient’s overall survival (OS) and improve their risk stratification.

**Methods:**

PRIMAGE database, including 513 patients (discovery cohort), was used for model training, validation, and testing. Additional 22 patients from different hospitals served as an external independent cohort. Primary tumor segmentation on T2-weighted MR images was semi-automatically edited by an experienced radiologist. From this area, 107 radiomics features were extracted. For the development of the prediction model, radiomics features were harmonized following the nested ComBat methodology and nested cross-validation approach was employed to determine the optimal preprocessing and model configuration.

**Results:**

The discovery cohort yielded a 78.8 ± 4.9 and 77.7 ± 6.1 of C index and time-dependent area under de curve (AUC), respectively, over the test set, with a random survival forest exhibiting the best performance. In the independent cohort, a C-index of 93.4 and a time-dependent AUC of 95.4 were achieved. Interpretability analysis identified lesion heterogeneity, size, and molecular variables as crucial factors in OS prediction. The model stratified neuroblastoma patients into low-, intermediate-, and high-risk categories, demonstrating a superior stratification compared to standard-of-care classification system in both cohorts.

**Discussion:**

Our results suggested that radiomics features improve current risk stratification systems in patients with neuroblastoma.

## 1 Introduction

Neuroblastoma (NB) is the most frequent solid cancer of early childhood, accounts for 7%–10% of all childhood cancers (1–3), and significantly benefits from imaging at every step of the patient journey. Most NB cases are diagnosed before the age of 5 years, and the median age at diagnosis is 22 months (4). Significant heterogeneity in tumor features and patient outcomes define NB (5–7), with approximately 60-70% of the cases being metastatic at presentation, usually in lymph nodes, liver, bone, and bone marrow (4). Due to the large clinical and biological divergency of NB, several staging systems have been created for risk stratification of patients.

At present, two major systems are used: The International Neuroblastoma Staging System (INSS) and the International Neuroblastoma Risk Group (INRG) staging system. The INSS, developed in 1986, is a postsurgical system that classifies patients according to the disease location, lymph node status, and extent of surgical resection (8, 9). The INRG, created in 2005, has largely replaced INSS, with the aim of stratifying patients regardless of surgical resection. It incorporates the presence of image defined risk factors (IDRF) to categorize locoregional tumors as L1 (IDRF absent) or L2 (IDRF present) and the presence of metastasis confined to special location (bone marrow, liver and/or skin) in children younger than 18 months as MS or any metastasis as M which is different from the MS definition (10, 11). As a result, the majority of current therapeutic strategies rely on INRG risk classification scores combining several clinical, imaging, pathologic, and genetic traits that have been linked to survival. This applies for the original INRG Classification System (10), and the revised version in 2021 by the Children’s Oncology Group (COG) (12), that classifies patients into low-, intermediate-, and high-risk groups (12, 13) based on their INRG stage, age at diagnosis, histology, and presence of molecular and pathologic biomarkers, such as MYCN amplification status, DNA ploidy, and segmental chromosomal aberrations. Treatment options and survival outcomes largely differ between risk groups, with low-risk patients experiencing a 5-year overall survival rate of 98% with no or minimal treatment compared to 62% of high-risk patients (12) despite an intense treatment.

The complex biological and clinical heterogeneity inherent in NB foster the development of more accurate prognostic markers and improved survival prediction tools at diagnosis to refine patient stratification and better tailor treatments. This could be especially relevant for patients with poor prognosis and high risk, who would be ideal candidates for treatment intensification strategies and close monitoring. In recent years, artificial intelligence (AI) has generated high expectations for improving cancer diagnosis, prognosis, and therapy, with machine learning approaches bringing exciting progress in digital pathology and diagnostics, and enriching foundational and drug-discovery research (14). Radiomics, the extraction of mineable data from medical images that allow tumor heterogeneity and phenotypic assessments (15), has opened up new avenues for clinical outcome prediction when combined with AI-based methods (16).

For AI radiomics models to achieve generalizability towards predicting clinical endpoints in oncology, the creation of international high-quality multi-omics registries is essential, especially in diseases with a low incidence, such as NB (2.9 cases per million children) (17). These real-world data repositories foster collaboration and facilitating a deeper understanding of the intricacies of oncological conditions with low prevalence. In this context, the PRIMAGE (PRedictive In-silico Multiscale Analytics to support cancer personalized diaGnosis and prognosis, Empowered by imaging biomarkers) (18) EU-funded project was conceived for the development of computational analysis methods of medical images applied to childhood cancer. This initiative has culminated in the largest and highest quality database of NB in Europe, with a total of 1,138 patients integrating imaging data alongside diagnostic, treatment, and outcome information.

In this work, we aimed to develop a machine learning-based model for the prediction of overall survival (OS) and risk assessment in children with NB within the PRIMAGE project.

## 2 Materials and methods

### 2.1 Dataset creation

The dataset used for model’s development was a subset of PRIMAGE patients (18), consisting of patients diagnosed between 2002 and 2021 who participated in the SIOPEN trials (19, 20). The inclusion criteria were as follows: 1) availability of a transversal T2-weighted (T2w) MR imaging series, with or without fat suppression including the primary tumor; 2) availability of clinical and molecular data; and 3) patient’s OS defined as the time between diagnosis and either death or the last available follow-up.

**Figure 1:**
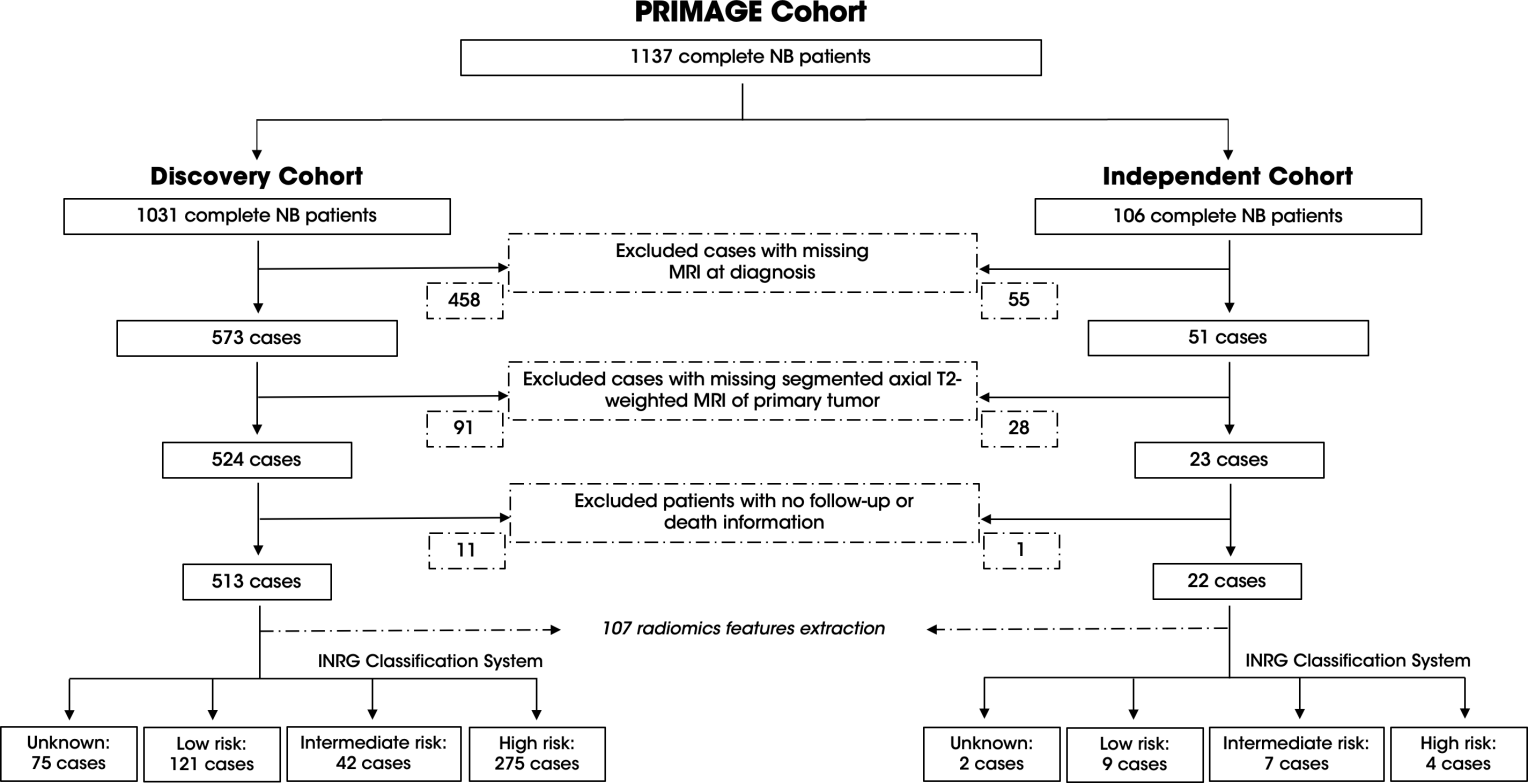
Overview of the PRIMAGE Cohort and patient selection process for the discovery and independent cohorts. The consort diagram details the inclusion and exclusion criteria applied to the 1137 NB patients in the PRIMAGE cohort. The discovery cohort (left) included 513 patients after excluding cases with missing information. The independent cohort (right) comprised 22 patients after applying the same criteria. The INRG classification system of each cohort is provided with the corresponding case numbers.

Two different cohorts of patients were divided to develop the machine learning model, the discovery cohort for model training, validation, and testing; and the independent cohort, to validate the final model in a population from different centers. The discovery cohort was composed of 1,032 patients with NB, of whom 524 had available transversal T2w MRI exam of the primary tumor, with or without fat suppression. From the discovery cohort, 11 of 524 patient, were excluded due to missing information of follow-up or death. The independent cohort consisted of 106 patients, of which 23 met the specified inclusion criteria. From the independent cohort, one patient was excluded due to the lack of follow-up data. Finally, 513 patients for the discovery cohort and 22 cases for the independent cohort were included. The complete process is summarized in **Figure 1** which specifies the number of patients excluded in each step.

#### 2.1.1 Image analysis and pre-processing

MR examinations were obtained from multiple hospitals and scanners, with different acquisition protocols. **Table 1** provides an overview of the MR parameters from both discovery and independent cohorts.

**Table 1:** Summary of MR acquisition parameters for the discovery and independent cohorts.

MR Acquisition Parameters	Discovery Cohort N = 513		Independent Cohort N = 22		p-value
	n (%)	Median [IQR]	n (%)	Median [IQR]	
*Manufacturer* *▪ Siemens* *▪ Philips* *▪ GE* *▪ Unknown*	274 (53.4) 125 (24.4) 83 (16.2) 31 (6.0)	–	6 (27.3) 11 (50.0) 5 (22.7) 0	–	0.071
*Magnetic field (T):* *▪ 1.5* *▪ 3*	423 (82.5) 90 (17.5)	–	22 (100) 0	–	1.000
*Echo time (ms)*	–	92.0 [80.0 – 103.0]	–	99.8 [86.1 – 108.3]	0.124
*Repetition time (ms)*	–	3180.4 [1600.0 – 4910.0]	–	2939.5 [2013.9 – 5591.0]	0.407
*Slice thickness (mm)*	–	4.0 [3.6 – 5.0]	–	4.0 [3.0 – 5.0]	0.971
*Pixel Spacing X (mm)*	–	0.74 [0.55 – 0.94]	–	0.64 [0.51 – 0.84]	0.187
*Pixel Spacing Y (mm)*	–	0.74 [0.55 – 0.94]	–	0.64 [0.51 – 0.84]	0.188

P-values for *Manufacturer* and *Magnetic field* were calculated with a chi-square test, while the Mann-Whitney U Test was used for the other numerical parameters due to the lack of normality.

MR, magnetic resonance; IQR, interquartile range.

To harmonized image quality, MR images underwent image denoising (21) through non-local means filter (22) and N4 bias field correction (23). Subsequently, spatial resampling was executed through b-splines interpolation to a common voxel size of 1x1x6 mm^3^. Finally, intensity normalization was applied through z-score normalization. Furthermore, during radiomics features extraction, a gray value discretization was applied, fixing the bin width of 5 to maintain a direct correlation with the original intensity scale.

#### 2.1.2 Primary tumor segmentation and radiomics features extraction

Before radiomics feature extraction, segmentation of the primary tumor was performed. A semi-automatic approach was employed through an AI-based NB segmentation model developed within the PRIMAGE project (24). The resulting segmentations underwent thorough examination and were edited by an experienced radiologist. Once images were prepared and segmented, 107 radiomics features were extracted using PyRadiomics (v3.0) (25) to obtain shape, first-order, and second-order features from the primary tumor. Both segmentation and radiomic extraction were performed on PRIMAGE platform, based on Quibim Precision (Quibim SL, Valencia, Spain) (26).

### 2.2 Database curation and feature engineering

The AI model was developed using clinical and molecular variables at diagnosis, together with radiomics features extracted from MRI scans. For this purpose, an initial pre-processing step was required, in which a curation of the clinical database and the harmonization of radiomics features were performed.

A careful selection of the most important variables within the PRIMAGE platform was undertaken, adhering to the criteria set forth by clinical and molecular experts. Normalization of lactate dehydrogenase (LDH) was performed based on each patient’s respective normal value. New variables were generated, based on the data available in the PRIMAGE platform, to precisely indicate tumor location, the presence of clinical symptoms, and the results of bone marrow tests. Additionally, low-frequency categorical variables were combined and grouped into alternative categories, with a subsequent implementation of dummy encoding. The clinical and molecular variables incorporated in the model development are detailed in Supplementary Information 1.

Missing values were imputed during the training phase using the MICE (Multiple Imputation by Chained Equations) algorithm (27) for variables with less than 20% of missing data. Each missing value was imputed three times, with the final value being the median or the mode for numerical and categorical variables, respectively. For variables with 20-30% missing values, such as tumor histology type and tumor differentiation grade, reliable imputation was not feasible. Consequently, these variables were instead dummy encoded, handling missing values as an additional class which was subsequently excluded from analysis. Variables exceeding 30% missing data, such as INRG staging system were discarded.

#### 2.2.1 Data harmonization

For radiomics features harmonization, Nested ComBat methodology was employed to identify the optimal approach for correcting the two main batch effects: MR scanner manufacturer and magnetic field. This methodology provides a sequential workflow for radiomics features harmonization to compensate for the multicenter heterogeneity caused by multiple batch effects (28). The batch effects were identified using a Cramer’s V and Theil’s U test.

Differences in radiomics features before and after ComBat harmonization were assessed with statistical tests and effect size measures. Discrepancies were considered significant if they were accompanied by a p-value < 0.05 and an effect size that was at least of a medium magnitude. For variables following a normal distribution, the t-test supplemented with Cohen’s D (medium effect >= 0.5), and the ANOVA test complemented by eta squared (medium effect >= 0.06) were employed. In the case of variables that deviated from normality, the Mann-Whitney U test along with a common language effect size (medium effect >= 0.3) and the Kruskal-Wallis test paired with eta squared (medium effect >= 0.06) were utilized. For variables with medium and large effect sizes, it was determined that these effects were due to outliers in the original variables that could not be corrected through harmonization, leading to their exclusion from the analysis.

### 2.3 Model development

A nested cross-validation was applied as training methodology with a 5x5 configuration for the development of the OS prediction model, maximizing the concordance index (C index). To avoid introducing bias when performing the cross-validation splits, each partition was equitably stratified by the INRG classification system and the patients’ censored status. In addition, it was assessed that there were no significant differences in any of the partitions for some of the most important clinical variables such as age, MYCN status, sex, and INSS staging, employing a t-test or ANOVA for numerical variables, and a chi-square test for categorical variables.

**Figure 2:**
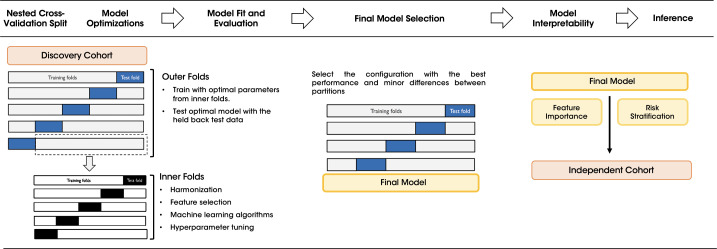
Workflow of the nested cross-validation scheme for training and validating the OS model. The diagram illustrates the step-by-step nested CV process applied to the discovery cohort to optimize the model configuration. The outer folds were used to test the optimal parameters determined in the inner folds. The best configuration was selected as the final model and its feature importance and risk stratification were assessed before making inference in the independent cohort.

In the inner loop training phase, the harmonization step was applied to the test/validation partition using transfer learning ComBat (29). For feature selection, two approaches were tested: a univariate Cox model which ranks the most informative features and the maximum relevance minimum redundancy (MRMR) algorithm (30). Finally, the state-of-the-art machine learning algorithms were assessed for survival prediction, including Cox’s proportional hazards model, random survival forest, extra random survival trees, and XGBoost survival embeddings (31, 32). This internal cross-validation was conducted automatically with the Optuna framework (v3.1) (33) for the hyperparameters optimization, combining all feature selection methods with different number of variables, with the machine learning models to identify the best-performing pipeline. Since the nested cross-validation generates different model configurations for each outer loop, the configuration with the best performance and minor differences between partitions was selected as the final model, see **Figure 2**.

Models were trained and validated in the discovery cohort and subsequently, once the final model was selected, tested in the external independent cohort. For model’s interpretability the SHAP (SHapley Additive exPlanations) values were calculated to show the relationships established by the model when making predictions, both at a model level and at patient level to ensure explainability in decision-making processes (34).

Finally, to provide the risk stratification, a set of thresholds were defined to classify patients into three groups based on their OS predicted probabilities: low, intermediate, and high risk. The classification thresholds were determined by optimizing the differences between the three survival groups in the training partitions via a *LogRank* test.

## 3 Results

### 3.1 Patient characteristics

Baseline patient curated characteristics, including selected clinical and molecular variables after model development, are summarized in **Table 2**. There were no significant differences observed in the distributions of these variables between the discovery and independent cohorts, and OS was also comparable, as shown in the Kaplan-Meier curves in the **Supplementary Figure 1**. The statistical differences between the discovery cohort and the patients without MRI studies were also assessed (**Supplementary Table 1**). Differences between both cohorts were found for the normalized values of LDH and for age. However, in the case of the former, normalization ensured consistency in the model’s performance. Similarly, both cohorts had a median age above the 18-month clinical evaluation threshold, indicating they represented similar patient populations with poor prognosis and higher metastatic risk.

**Table 2:** Clinical and molecular data for the discovery and independent cohorts showing balanced distributions.

Characteristics	Discovery Cohort N = 513		Independent Cohort N = 22		p-value
	n (%)	Median [IQR]	n (%)	Median [IQR]	
*Sex* *▪ Male* *▪ Female*	264 (50.5) 259 (49.5)	–	14 (63.6) 8 (36.4)	–	1.000
*Age at diagnosis (months)*	–	22.0 [8.9 – 43.0]	–	23.0 [10.0 – 31.5]	0.994
*LDH normalized*	–	1.5 [0.91 – 3.1]	–	1.7 [1.2 – 3.2]	0.320
*MYCN* *▪ Amplified* *▪ No amplified* *▪ Missing data*	113 (21.6) 371 (70.9) 39 (7.5)	–	2 (9.1) 17 (77.3) 3 (13.6)	–	1.000
*Risk group INRG* *▪ Low* *▪ Intermediate* *▪ High* *▪ Missing data*	125 (23.9) 42 (8.0) 281 (53.7) 75 (14.3)	–	9 (40.9) 7 (31.8) 4 (18.2) 2 (9.1)	–	0.847
*Staging INSS* *▪ 1* *▪ 2/3* *▪ 4* *▪ 4s* *▪ Missing data*	25 (4.7) 157 (30.0) 289 (55.3) 37 (7.1) 15 (2.9)	–	2 (9.1) 11 (50.0) 5 (22.7) 0 4 (18.2)	–	0.699
*Grade of differentiation of the tumor* *▪ Undifferentiated* *▪ Poorly differentiated* *▪ Differentiating* *▪ Missing data*	41 (7.8) 242 (45.3) 42 (8.0) 198 (37.9)	–	2 (9.1) 10 (45.4) 4 (18.2) 6 (27.3)	–	0.136
*Histology type of the tumor* *▪ Neuroblastoma* *▪ Ganglioneuroma or intermixed ganglioneuroblastoma* *▪ Missing data*	413 (71.0.) 34 (6.5) 76 (14.5)	–	19 (86.4) 2 (9.1) 1 (4.5)	–	1.000
*Bone marrow results* *▪ Positive* *▪ Negative*	271 (51.8) 252 (48.2)	–	5 (22.7) 17 (77.3)	–	0.321
*Clinical symptoms* *▪ Positive* *▪ Negative* *▪ Missing data*	244 (46.7) 207 (39.5) 72 (13.8)	–	10 (50.0) 11 (45.4) 1 (4.6)	–	0.189
*Tumor location: abdomen* *▪ Positive* *▪ Negative* *▪ Missing data*	390 (74.6) 112 (21.4) 21 (4.0)	–	10 (50.0) 11 (45.4) 1 (4.6)	–	0.781
*Tumor location: other location* *▪ Positive* *▪ Negative* *▪ Missing data*	163 (31.2) 311 (59.5) 49 (9.3)	–	13 (59.1) 9 (40.9) 0	–	0.230

P-values for categorical features were calculated with a chi-square test, while the Mann-Whitney U Test was used for numerical variables due to the lack of normality.

INRG, International Neuroblastoma Risk Group Classification System; INSS, International Neuroblastoma Staging System; IQR, interquartile range; LDH, lactate dehydrogenase.

### 3.2 Radiomics features extraction and harmonization

A total of 107 radiomics features were extracted from the primary tumor delineated over the T2w MR images and harmonized. To address the two batch effects, MR manufacturer differences and magnetic field strength, both combinations were evaluated. The combination that minimized the number of radiomic features with significant differences, following the nested ComBat methodology, was chosen as the harmonization pipeline.

The optimal sequential harmonization process was defined as a first step for MR manufacturer and then magnetic field strength, yielding the greatest reduction in significant differences, see **Table 3**. As a result, the number of variables showing differences were reduced to 12 for the MR manufacturer and to 7 for the magnetic field. Most variables continued exhibiting differences post-harmonization had a small effect size, indicating minimal influence. Finally, 10 features were excluded from the analysis due to medium and larger effect sizes measures, see **Supplementary Table 2**. Therefore, a total of 97 radiomics features were finally inputted to the ML models.

**Table 3:** Number of radiomics features with differences before and after harmonization caused by the batch effects: manufacturer and magnetic field.

Original Differences by Batch Effect (n)		Radiomics Features with Differences after Harmonization (n)	
*Manufacturer*	72	Harmonization Pipeline 1: Manufacturer + Magnetic Field	
		Differences by Manufacturer	12
		Differences by Magnetic Field	7
*Magnetic Field*	31	Harmonization Pipeline 2: Magnetic Field + Manufacturer	
		Differences by Manufacturer	14
		Differences by Magnetic Field	19

Nested Combat harmonization was applied to correct possible differences caused by the manufacturer and magnetic field batch effects for radiomics features. The optimal harmonization pipeline was Manufacturer + Magnetic Field, which minimized the number of significant differences.

### 3.3 OS prediction model and risk stratification

After the nested cross-validation, the random survival forest with a set of eight features emerged as the top-performing model for the overall survival prediction with a C index and a time-dependent AUC of 78.8 ± 4.9 and 77.7 ± 6.1 (mean ± standard deviation), respectively, in the test sets of the discovery cohort. The model performance was tested in the independent cohort, where an improved C index and time-dependent AUC of 93.4 and 95.4 were obtained. **Table 4** provides a summary of the model metrics in both cohorts.

**Table 4:** Random survival forest performance evaluation.

Evaluation	Random Survival Forest Performance			
	Discovery Cohort (Test)		Independent Cohort	
*C index*	78.8±4.9		93.4	
*Time-dependent AUC*	77.7±6.1		95.4	
Brier’s score*	12.5±0.9		15.7	
Baseline for reference**	25.2±2.9		-	
*LogRank test (p-value)*	RSF model (n)	INRG (n)	RSF model (n)	INRG (n)
*Low vs Intermediate*	0.48 (39 vs 43)	0.43 (25 vs 16)	0.12 (13 vs 7)	0.43 (9 vs 7)
*High vs Low*	<0.005 (21 vs 39)	0.05 (62 vs 25)	<0.005 (2 vs 13)	0.56 (4 vs 9)
*High vs Intermediate*	<0.005 (21 vs 43)	0.05 (62 vs 16)	<0.005 (2 vs 7)	0.90 (4 vs 7)

*Lower is better. **Random prediction model as reference.

INRG, International Neuroblastoma Risk Group Classification System; RSF, random survival forest.

On top, model metrics on the test partition in the discovery cohort and in the independent cohort. At bottom, *LogRank* test *p*-values for Kaplan-Meier curves based on the INRG classification system and random survival forest stratification for both cohorts.

The random survival forest model assigned a risk score to each patient, allowing classification into three survival groups low, intermediate, and high risk based on their OS probabilities. The classification thresholds were optimized in the training partition using a *LogRank* test to maximize differences between the groups: patients with predicted risk scores below 6.3 were classified as low risk, those with scores above 16.1 as high risk, and those with intermediate scores as intermediate risk.

**Figure 3:**
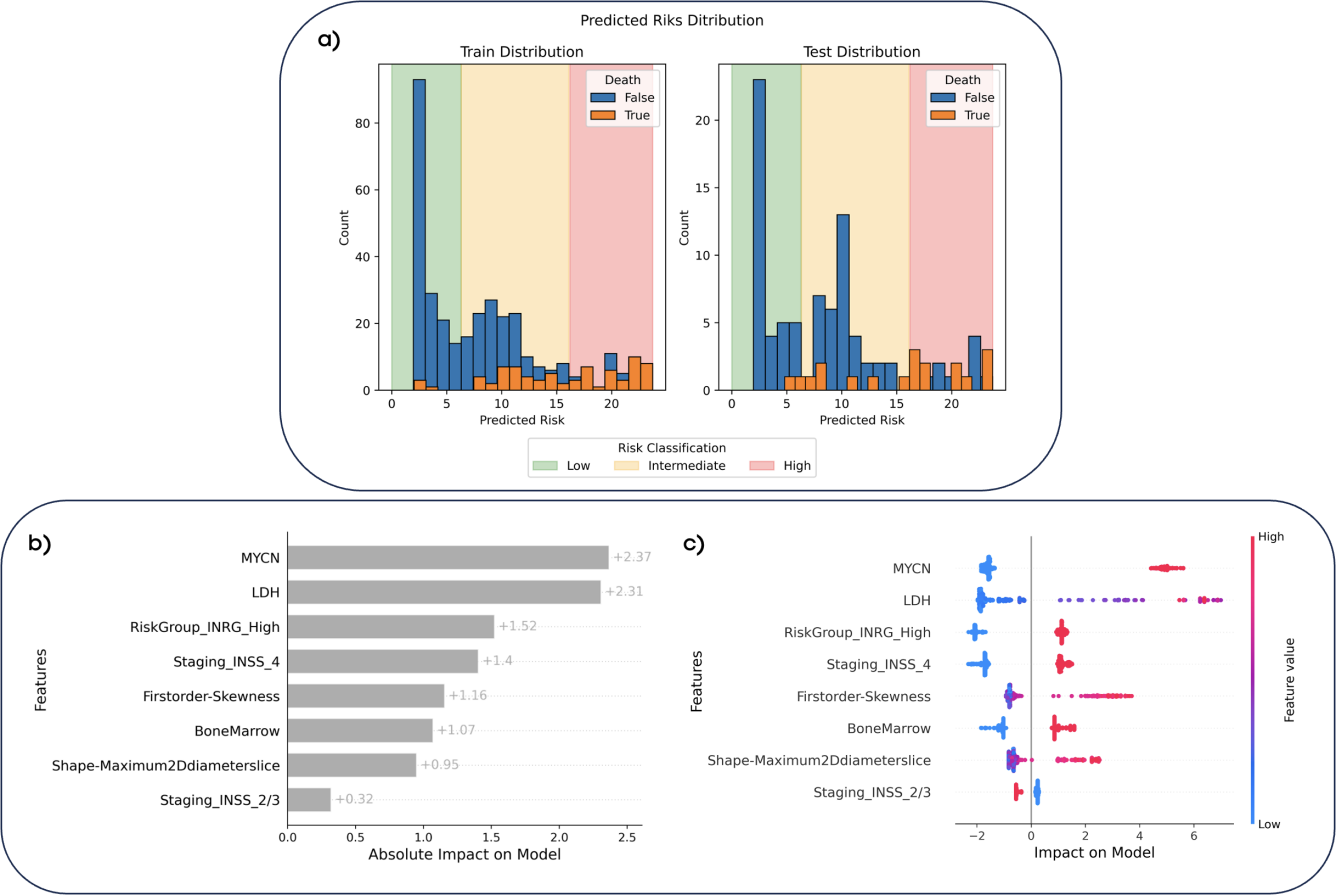
Predicted risk distributions and interpretability of the random survival forest model. **(A)** The predicted risk distributions for the training and test datasets, stratified into low-risk (<6.3, green), intermediate-risk (6.3–16.1, yellow), and high-risk (>16.1, red) categories based on the model’s risk scores. Bars represent the counts of patient who survived (blue) and those who died (orange) according to the ground truth. **(B)** Feature importance with SHAP values, showcasing the absolute impact of each feature on the model’s predictions. **(C)** SHAP dependence plot showing the relationship between feature values and their impact on risk predictions. Red markers indicate high feature values, while blue markers represent low values. Positive SHAP values indicate a higher predicted risk, while negative values indicate lower risk.

**Figure 3A** illustrates the predicted risk distribution of patients in both the training and test sets of the random survival forest model, showcasing the thresholds selected during training and applied to the test set. Most patients who died (orange) were observed to fall within the intermediate-risk (yellow) and high-risk (red) groups, demonstrating the model’s ability to discriminate between patient risk levels. In addition, **Figures 3B, C** show the interpretability analysis with SHAP values. It is observed that this model consisted of a combination of clinical variables and radiomics features. The most important variable was MYCN status, closely followed by LDH value, see **Figure 3B**. Positive values of MYCN (i.e., MYCN amplification) or very high values of LDH aligned with high-risk. Regarding radiomics variables, the model included the skewness, which measures the asymmetry of the distribution of voxel intensities of the primary tumor about the mean value (highly heterogeneous tissues show higher absolute skewness than homogeneous ones), and the maximum 2D diameter, which is related with tumor size. For both variables, higher values of these features were associated with a higher risk, see **Figure 3C**.

### 3.4 Comparison with INRG classification system

**Figure 4:**
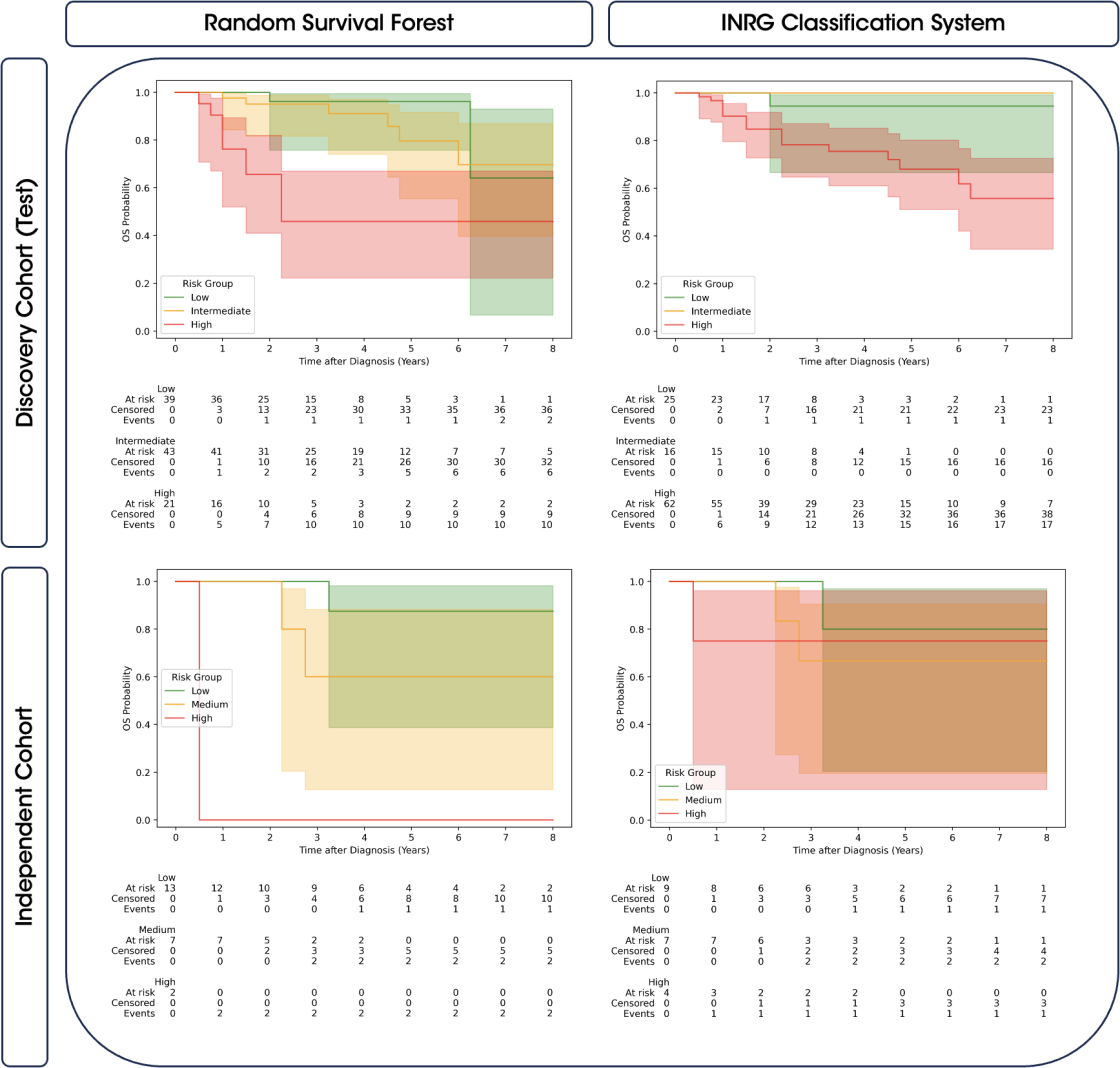
Comparison of Kaplan-Meier survival curves with 95% confidence intervals for predicted risk groups and INRG. The survival curves are stratified by the predicted risk groups provided by the random survival forest model (left) and the INRG classification system (right). Results are shown for both the discovery cohort (top) and the independent cohort (bottom). The risk groups (low, intermediate, and high) are color-coded, and confidence intervals highlight the variability of the survival estimates.

The stratification capability of the random survival forest model was compared to that offered by the INRG classification system, assessing the Kaplan-Meier curves with a *LogRank* test of the different risk groups in the final model test set of the discovery cohort and in the independent cohort, see **Figure 4** and **Table 4**.

As observed in **Table 4**, Kaplan-Meier curves obtained from the random survival forest model for the high-risk group demonstrated significant differences when compared to the low- and intermediate-risk groups in both cohorts. However, no significant differences were observed between the low and intermediate curves. On the other hand, the INRG classification system bordered on significance for distinguishing high-risk group with low- and intermediate-risk groups in the discovery cohort and did not provide significant differences across all risk group comparisons in the independent cohort. Visually, **Figure 4** also showed a greater overlap between the confidence intervals of the high-risk group and the other groups in the INRG, whereas the random survival forest model exhibits less overlap, particularly in the independent cohort.

## 4 Discussion

The integration of AI methodologies with clinical research has become increasingly significant. This study examines the efficacy of a random survival forest model developed to improve stratification in NB patients, highlighting the potential of radiomics features to enhance existing risk stratification systems.

Thus, our model successfully captured meaningful relationships, enabling accurate predictions with a C index and a time-dependent AUC of 78.8 ± 4.9 and 77.7 ± 6.1 in the discovery cohort, and a value of 93.4 and 95.4 in the independent cohort. The comparison of stratifications revealed that the random survival forest model was able to effectively discriminate patients at high-risk from those at low- and intermediate-risk. This discriminatory capability was significant, particularly when compared to the INRG classification system, which partially failed to achieve effective discrimination in both the discovery and validation cohorts, as indicated in **Table 4**. Consequently, our results of a model incorporating standard clinical, molecular, and radiomics information suggest a better patient stratification system in comparison to the existing clinical standard.

Importantly, the interpretability analysis of this model revealed that clinical and molecular variables played a pivotal role in the prediction, with radiomics variables serving as complementary fine-tuning support. Thus, the random survival forest model relied on a set of eight predictive variables, three laboratory biomarkers (MYCN, LDH, and bone marrow test results), three subclasses of different staging systems from INSS and INRG, and two radiomic variables (skewness and 2D maximum diameter) from the multitude of feature combinations automatically evaluated during the training process. The most influential variable was MYCN status, closely followed by LDH value. MYCN amplification and very high values of LDH favored predictions of higher risk. Regarding variables with a moderate impact on the model’s output, clinical variables such as INRG high-risk group, INSS stage 4 and bone marrow positive values indicated a higher risk prediction. Conversely, 2/3 INSS staging tended to suggest a lower risk for patient prediction. It is important to note that all those clinical and molecular variables are interconnected and demonstrate a degree of correlation. Hence, MYCN is a very significant factor in defining a high-risk patient according to the INRG criteria, and together with elevated levels of LDH, both have been reported as important risk factors (10). Additionally, the majority of patients with bone marrow involvement exhibit metastases, which is one of the conditions for classifying a patient at stage 4 of the INSS (8). These factors are recognized as critical risk determinants, reinforcing the model’s dependence on them for accurate risk stratification in NB.

Regarding radiomics variables, *skewness* and *maximum 2D Diameter Slice*, which reflect heterogeneity and tumor size, respectively, exhibited a more complex behavior. In both cases, very high values contributed to increased risk, while intermediate and lower values contributed to risk reduction. This indicates that more heterogeneous and larger lesions are correlated with predictions of higher risk for patients. From a clinical perspective, lesion heterogeneity is one of the most critical factors in neuroblastoma research, as it has been hypothesized that more heterogeneous lesions are also the most aggressive (35). Therefore, the selection of a variable of this nature in the final model further supports this hypothesis through empirical data.

Notably, the relationships identified by the random survival forest model among clinical, molecular, and radiomic variables are consistent with those established by the current criteria (10). In this way, the staging systems serve as a foundation for the additional contribution provided by the rest of variables when generating predictions for each patient. This integration highlights the practicality of the model for real-world applications, offering a streamlined approach to patient stratification. The application of the model in clinical practice could be straightforward, requiring only laboratory variable results (estimated time 2-4 weeks). Staging could be inferred directly from the images, similar to radiomics, which could be extracted from lesions segmented automatically or semi-automatically (24).

A recent review on employing machine learning techniques in NB prediction models revealed the minimal application of radiomics in this field, with no predictive models incorporating radiomics for OS prediction or patient stratification (36). This highlights a critical gap in NB research, which our study aims to address. Existing studies combining these approaches have predominantly focused on predicting specific variables, such as bone marrow involvement (37, 38)and MYCN amplification (39), primarily using CT and, to a lesser extent, MR images. Studies predicting broader outcomes, such as the presence of metastases, grade of differentiation, or mortality, remain rare (38). While these studies report positive outcomes, their objective differ from ours, as they do not primarily focus on OS prediction and risk stratification. Furthermore, these studies typically involve smaller patient cohorts and lack independent validation analysis, limiting their generalizability compared to our approach. However, recent research has started to address these shared objectives, focusing on improving risk stratification in NB patients through innovative methodologies. For instance, a study utilizing multi-omics data, such as gene expression and copy number alterations (CNA), has demonstrated improved stratification within a super high-risk group validated in two separate datasets (40). This is in line with other studies in which emerging genomic biomarkers, such as BDP1 variants I1264M and V1347M, have shown potential in enhancing clinical outcome predictions in NB patients (41). Interestingly, another recent study has employed a similar approach to ours, using a random survival forest model based on intratumoral microbial gene abundance data extracted from RNA-seq to enhance risk stratification (42). This method identified subgroups with significant differences in survival and improved stratification within the evaluated sample compared to the COG classification. However, the sample size was again limited, with 120 patients and no external validation. Thus, our study represents an important advancement by using a significantly larger patient cohort than prior studies, incorporating a preliminary external independent validation set, and merging radiomics with additional clinical NB parameters. These contributions not only address existing gaps, but also open avenues for including different -omics variables to potentially enhance stratification in the future.

Our study has, however, some limitations, one of which is the size of the cohorts employed. Thus, the discovery cohort, with over 500 patients, can be considered small when compared to the current standards, such as the INRG (10), which is based on data from more than 8,800 patients. It is worth mentioning that to address this limitation and to extract the most valuable information from the data, our cohort was split; a nested cross-validation methodology with 5x5 folds was applied to ensure robust training and testing, enabling us to assess performance across the entire dataset. On the other hand, the relatively small size of the external independent cohort, with only 22 patients meeting all inclusion criteria, could be also identified as a limitation. However, it is important to emphasize that the results obtained in this cohort were very promising, with a C Index of 93.4 and good patient stratification. Nevertheless, these findings should be considered a preliminary assessment due to the limited sample size. Increasing the number of patients with additional external prospective cohorts from different institutions would help enhance the statistical power of the analysis and validate the robustness of the findings. Another limitation concerns the distribution of patients across the different risk groups. Specifically, more than 50% of the patients in the discovery cohort were classified as high-risk according to the INRG system, while the intermediate-risk class was underrepresented with only 8% of cases. This imbalance may have introduced a bias in the model towards the high-risk group, potentially resulting in a higher baseline risk. Consequently, some patients classified as high-risk by INRG might have been categorized into an intermediate-risk group by the model, which may have hindered the model’s ability to accurately discriminate these patients. However, the classification of high-risk patients using this model could help identify super high-risk patients relative to the INRG scale by pinpointing the subgroup of patients exhibiting the most pronounced decline in the survival curves. Including more low- and intermediate-risk patients in the discovery cohort would likely improve the stratification, by allowing the model to better learn the characteristics of these groups. Overall, there is a necessity of future studies involving larger and more diverse external datasets to confirm and refine the model’s performance across broader and more representative patient populations. It is also important to note that the treatment was not considered in the development of the models. As treatment may change throughout a patient’s disease progression onto second-line treatment, this could have contributed to variations in patient survival times. Finally, radiomics features were exclusively extracted from intra-tumoral regions, disregarding the potentially predictive relevance of the peri-tumoral zones and adjacent organs. In future studies, it would also be of interest to include new -omics data that could further increase the model performance. This effort would significantly enhance confidence in interpreting the results, allowing for a more reliable assessment of the model’s performance. Despite these limitations, our study represents a significant step forward in advancing risk stratification for neuroblastoma patients, highlighting the potential or radiomics and machine learning in this setting.

In conclusion, the implemented random survival forest model integrating radiomic features with standard clinical and molecular variables enabled the successful and reproducible stratification of patients with NB. The model effectively stratified NB patients into low-, intermediate-, and high-risk categories, suggesting the potential of radiomics features to enhance existing risk stratification systems. Further external prospective validation is now imperative, as it holds the promise of providing additional evidence to advance patient care and information in the clinical decision-making for NB patients.

## Data Availability

Raw data for this study were generated at PRIMAGE platform, whose data are publicly available upon request to the corresponding author for innovation or research on EUCAIM (European Federation for Cancer Images) catalogue. Requests to access these datasets should be directed to https://catalogue.eucaim.cancerimage.eu/#/collection/PRIMAGE-1.
