# Supplementary material for: Risk stratification in neuroblastoma patients through machine learning in the multicenter PRIMAGE cohort
Source: Front Oncol. 2025 Feb 21;15:1528836. doi: 10.3389/fonc.2025.1528836 (PMC11886962; doi:10.3389/fonc.2025.1528836)
Supplement: Supplementary file 1 [file DataSheet1.docx]

# **Supplementary materials**

**Supplementary Information 1.** List of clinical variables included as input in the predictive models. In parenthesis the type of the variable.

- Sex (categorical).
- Age at diagnosis in months (numerical).
- LDH normalized by patient’s normal value (numerical).
- Histology type of the tumor: neuroblastoma / ganglioneuroma or intermixed ganglioneuroblastoma (categorical).
  - Neuroblastoma group includes patients with neuroblastoma and nodular ganglioneuroblastoma.
  - Ganglioneuroma or intermixed ganglioneuroblastoma group includes patients with ganglioneuroma, inntermixed ganglioneuroblastoma and mature ganglioneuroma,
- Grade of differentiation of the tumor: differentiated / poorly differentiated /undifferentiated (categorical).
- MYCN status: amplified /non-amplified (categorical).
- Risk group according to INRG classification system: high /intermediate / low (categorical).
- INSS staging: 1/ 2-3 / 4 / 4s (categorical).
- Bone marrow results: positive / negative. If either the aspirate or trephine results were positive, the variable was also marked as positive (categorical).
- Tumor location: a*bdominal* and *other* classes are not exclusive.
  - Abdomen: positive /negative. Whether there was a tumor in the abdominal region (categorical).
  - Other location: positive / negative. If there was a tumor in a different region from the abdominal such as cervical, pelvic, thoracic, or other locations (categorical).
- Clinical symptoms: positive / negative. Whether the patient experienced any of the spinal cord syndrome, Horner's syndrome, diarrhea, opsoclonus myoclonus syndrome (OMS), or other symptoms (categorical) collected in the PRIMAGE project.

**Supplementary Table 1.** Clinical and molecular data for the Discovery Cohort and patients without available MRI study (Excluded Cohort). P-values for categorical features were calculated with a chi-square test, while the Mann-Whitney U Test was used for numerical variables due to the lack of normality.

| Characteristics | Discovery Cohort  N = 513 | | Excluded Cohort  N = 549 | | P-value |
| --- | --- | --- | --- | --- | --- |
|  | n (%) | Median [IQR] | n (%) | Median [IQR] |  |
| Sex   - Male - Female | 264 (50.5)  259 (49.5) | - | 264 (57.5)  195 (42.5) | - | 0.930 |
| Age at diagnosis (months) | - | 22.0  [8.9 - 43.0] | - | 28.0  [13.1 - 47.4] | 0.003 |
| LDH normalized | - | 1.5  [0.91 - 3.1] | - | 2.2  [1.3 – 4.8] | < 0.005 |
| MYCN   - Amplified - No amplified - Missing data | 113 (21.6)  371 (70.9)  39 (7.5) | - | 138 (30.1)  292 (63.6)  29 (6.3) | - | 1.000 |
| Risk group INRG   - Low - Medium - High - Missing data | 125 (23.9)  42 (8.0)  281 (53.7)  75 (14.3) | - | 24 (5.2)  13 (2.8)  333 (72.5)  89 (19.5) | - | 0.758 |
| Stagging INSS   - 1 - 2/3 - 4 - 4s - Missing data | 25 (4.7)  157 (30.0)  289 (55.3)  37 (7.1)  15 (2.9) | - | 3 (0.8)  103 (22.4)  316 (68.8)  25 (5.4)  12 (2.6) | - | 0.966 |
| Grade of differentiation of the tumor   - Undifferentiated - Poorly differentiated - Differentiating - Missing data | 41 (7.8)  242 (45.3)  42 (8.0)  198 (37.9) | - | 57 (12.4)  175 (38.1)  19 (4.2)  208 (45.3) | - | 0.876 |
| Histology type of the tumor   - Neuroblastoma - Ganglioneuroblastoma or intermixed ganglioneuroblastoma - Missing data | 413 (71.0.)  34 (6.5)  76 (14.5) | - | 345 (75.2)  5 (1.1)  109 (23.7) | - | 1.000 |
| Bone marrow results   - Positive - Negative | 271 (51.8)  252 (48.2) | - | 259 (56.4)  200 (43.6) | - | 0.996 |
| Clinical symptoms   - Positive - Negative - Missing data | 244 (46.7)  207 (39.5)  72 (13.8) | - | 225 (49.0)  127 (27.7)  107 (23.3) | - | 0.089 |
| Tumor location: abdomen   - Positive - Negative - Missing data | 390 (74.6)  112 (21.4)  21 (4.0) | - | 380 (82.8)  36 (9.4)  43 (7.8) | - | 0.851 |
| Tumor location: others   - Positive - Negative - Missing data | 163 (31.2)  311 (59.5)  49 (9.3) | - | 87 (18.9)  189 (41.2)  183 (39.9) | - | 0.753 |
| Abbreviations: IQR, interquartile range; LDH, lactate dehydrogenase. | | | | | |

**Supplementary Table 2.** Features with significant differences and effect size after harmonization pipeline by manufacturer and magnetic field. Features with a large impact (highlighted) were excluded from the model.

| Batch Effect | Radiomic Feature | Effect Size |
| --- | --- | --- |
| Manufacturer | First order: Kurtosis | Small |
|  | GLCM: Cluster Shade | Small |
|  | GLCM: Joint Energy | Small |
|  | GLCM: Maximum Probability | Small |
|  | GLDM: Large Dependence Low Gray Level Emphasis | Small |
|  | GLRLM: Long Run Emphasis | Small |
|  | GLRLM: Run Variance | Small |
|  | GLSZM: Large Area Emphasis | Large |
|  | GLSZM: Large Area High Gray Level Emphasis | Small |
|  | GLSZM: Large Area Low Gray Level Emphasis | Large |
|  | GLSZM: Zone Variance | Large |
|  | NGTDM: Coarseness | Large |
|  | First order: Kurtosis | Small |
|  | GLCM: Cluster Shade | Small |
|  | GLCM: Joint Energy | Small |
|  | GLCM: Maximum Probability | Small |
|  | GLDM: Large Dependence Low Gray Level Emphasis | Small |
| Magnetic Field | GLCM: Cluster Prominence | Medium |
|  | GLDM: Small Dependence Low Gray Level Emphasis | Large |
|  | GLRLM: Long Run Emphasis | Large |
|  | GLRLM: Long Run Low Gray Level Emphasis | Large |
|  | GLRLM: Run Variance | Large |
|  | GLSZM: Large Area Low Gray Level Emphasis | Large |
|  | NGTDM: Coarseness | Large |


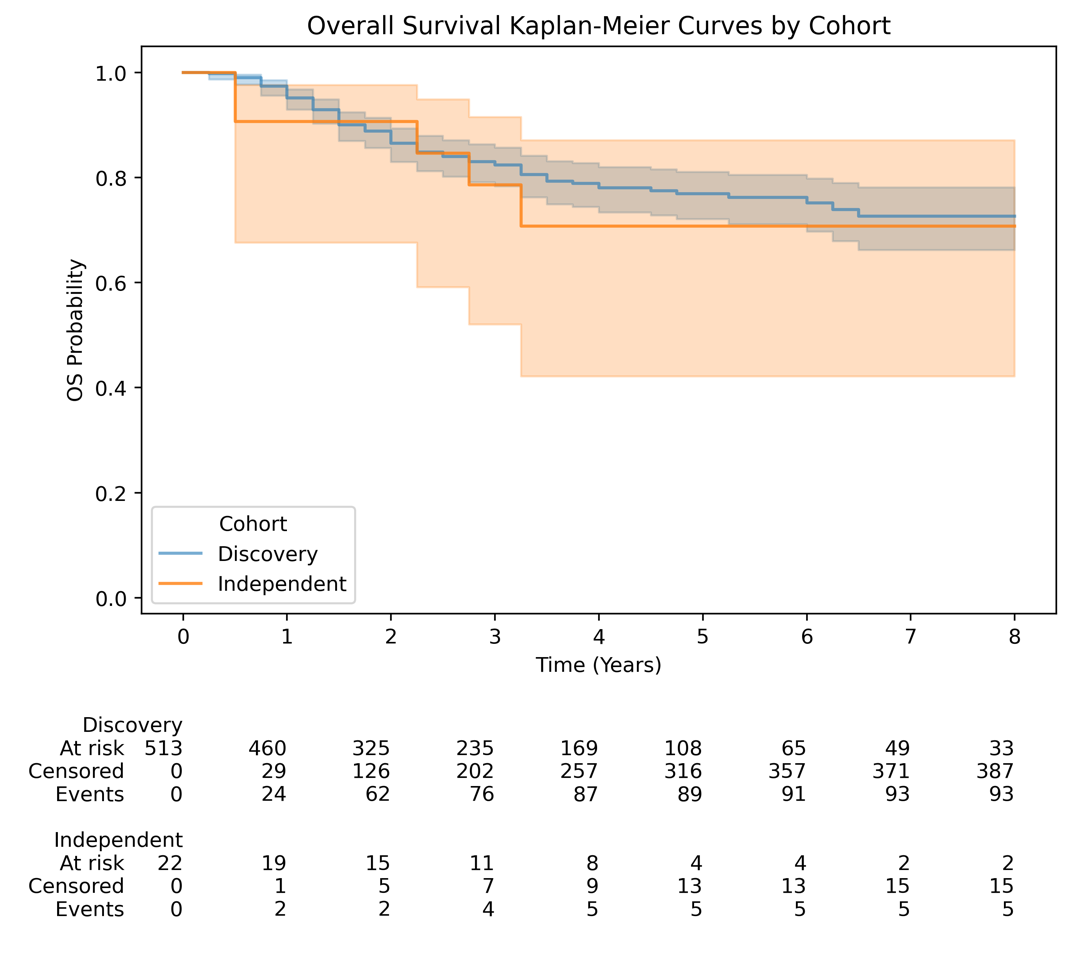


**Supplementary Figure 1**. OS Kaplan-Meier curves with 95% confidence intervals in both the Discovery (blue) and Independent (orange) cohorts. No significant differences were found in the *LogRank* test between the curves (p-value = 0.720).
